# Supplementary material for: Transcriptome Sequencing Reveals the Mechanism of Auxin Regulation during Root Expansion in Carrot
Source: Int J Mol Sci. 2024 Mar 18;25(6):3425. doi: 10.3390/ijms25063425 (PMC10970087; doi:10.3390/ijms25063425)
Supplement: Supplementary file 1 [file ijms-25-03425-s001.zip › Supplementary materials_Figures.pdf]

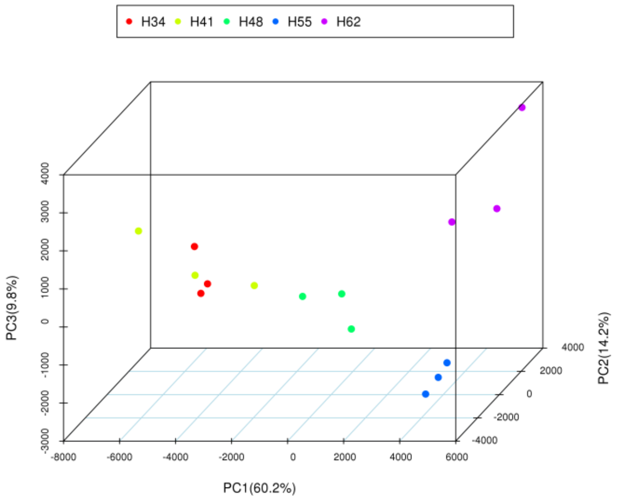

Figure S1. Principal component analysis diagram of the sample based in sequencing data. X, Y, and Z axes: different principal components, and different groups are distinguished by colors.

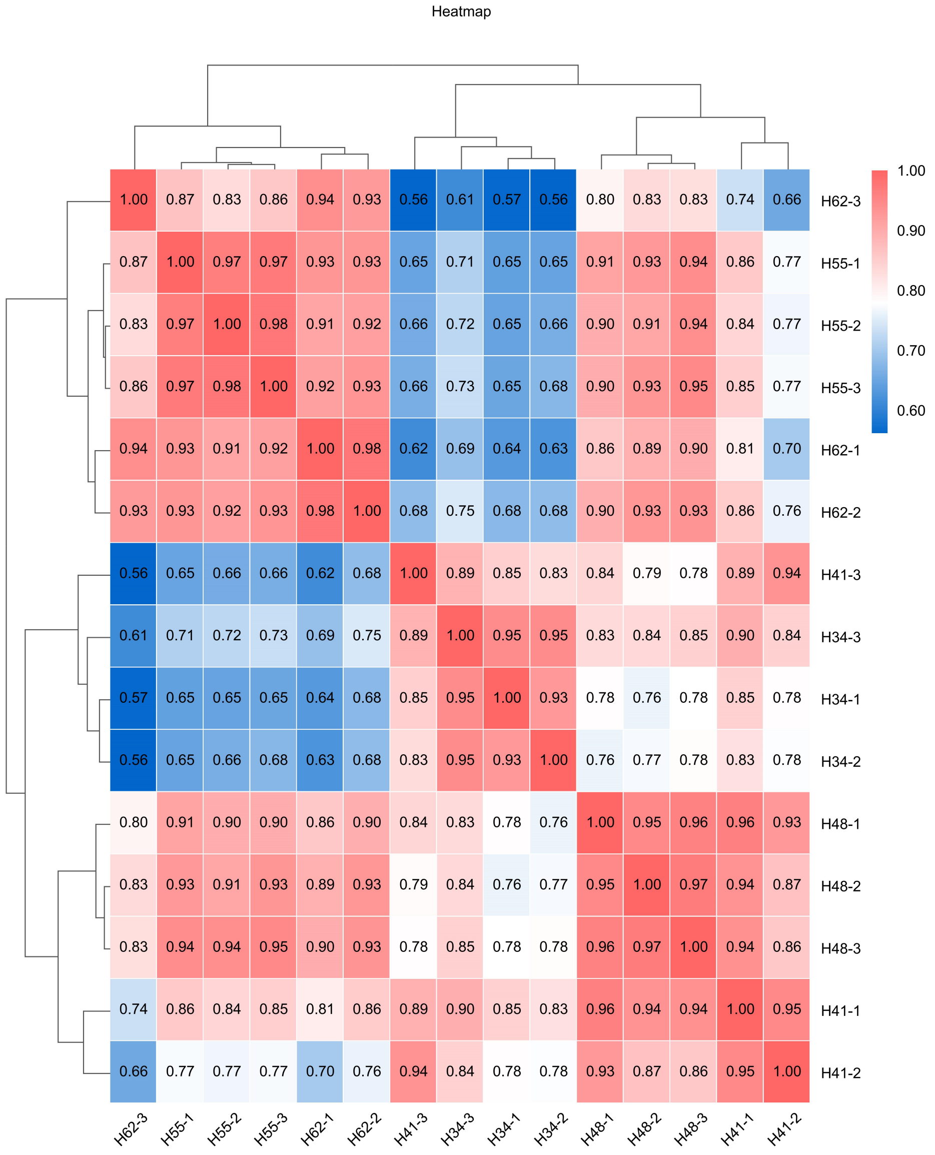

Figure S2. Correlation heat map of RNA sequencing data across the 3 biological replicates. H34, H41, H48, H55, and H62: 34, 41, 48, 55, and 62 days after sowing, respectively.

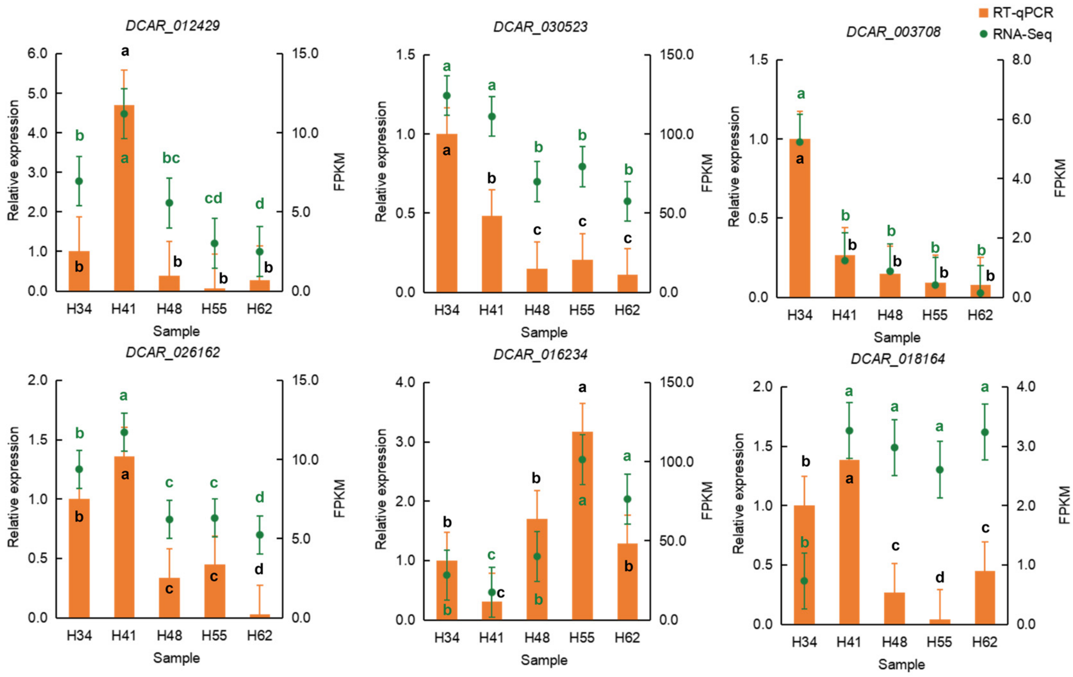

Figure S3. RT-qPCR verification of gene expression. Orange columns: relative expression of genes in RT-qPCR; Black letters: significant differences of RT-qPCR; Green dots: FPKM value of genes in RNA-seq; Green letters: significant differences of FPKM; *Duncan's* test, $p < 0.05$.

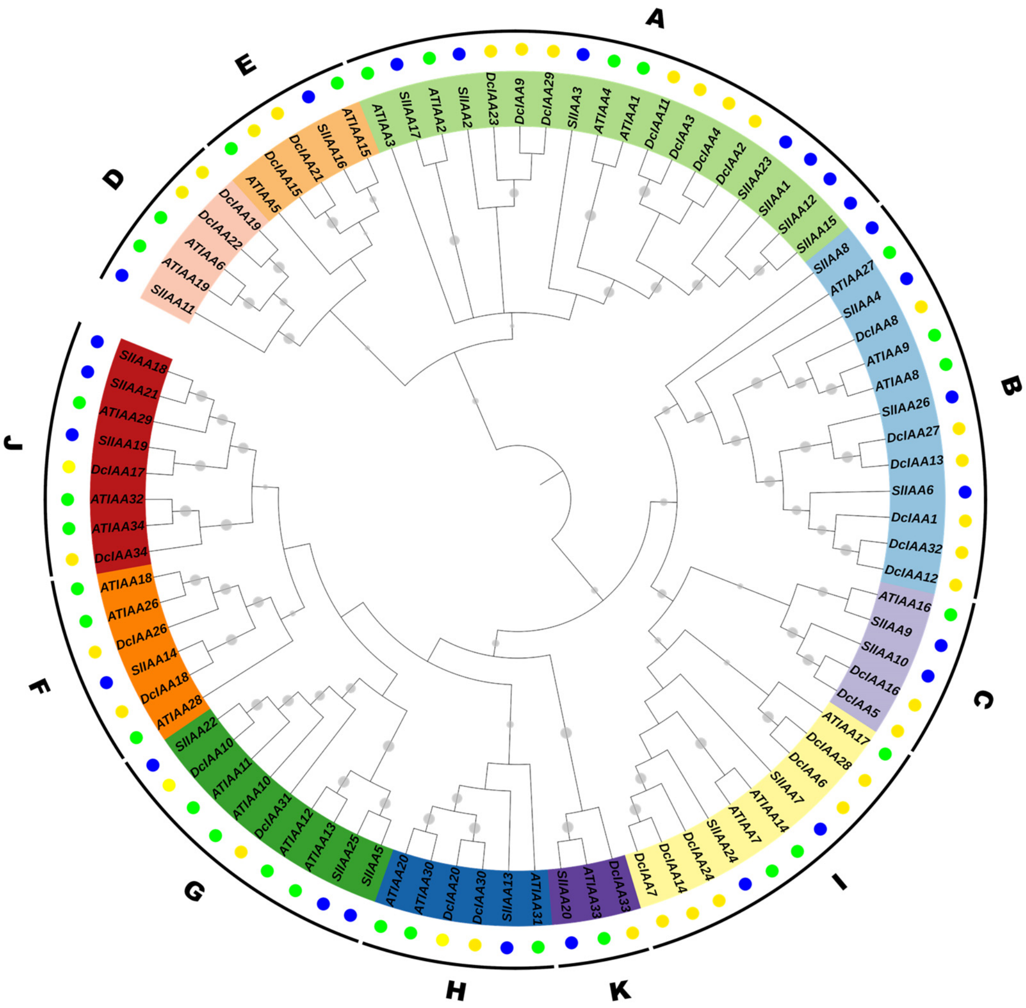

Figure S4. Phylogenetic analysis of *DcIAA* genes among different family members of carrot, tomato, and *Arabidopsis* shown in yellow, blue, and green dots, respectively. The outside circle of the phylogenetic tree shows different clades of the tree. The dots on the branch shown bootstraps value ≥ 70.
