# Supplementary material for: Transcriptome Sequencing Reveals the Mechanism of Auxin Regulation during Root Expansion in Carrot
Source: Int J Mol Sci. 2024 Mar 18;25(6):3425. doi: 10.3390/ijms25063425 (PMC10970087; doi:10.3390/ijms25063425)
Supplement: Supplementary file 1 [file ijms-25-03425-s001.zip › Supplementary materials_Table S1S4.pdf]

Table S1. Statistical table of sequencing results and reference genome sequence alignment

Samples names	Total reads	Mapped reads	GC content / %	Q30 / %
H34-1	50,482,792	43,904,121 (86.97%)	44.45	94.48
H34-2	46,875,676	41,326,527 (88.16%)	44.80	93.73
H34-3	41,411,862	36,349,465 (87.78%)	44.71	94.24
H41-1	47,988,102	42,380,689 (88.31%)	44.19	93.61
H41-2	54,648,016	48,176,207 (88.16%)	44.12	94.52
H41-3	62,422,836	54,907,550 (87.96%)	44.30	94.36
H48-1	52,478,140	45,856,626 (87.38%)	44.21	91.96
H48-2	49,544,826	43,291,921 (87.38%)	44.19	91.28
H48-3	43,021,704	37,670,526 (87.56%)	44.28	92.03
H55-1	51,076,736	44,997,147 (88.10%)	44.43	94.07
H55-2	51,194,918	45,261,649 (88.41%)	44.16	94.04
H55-3	44,755,398	39,547,258 (88.36%)	44.30	94.28
H62-1	42,864,510	37,499,341 (87.48%)	44.46	91.13
H62-2	43,901,792	37,499,341 (87.48%)	44.25	92.72
H62-3	38,794,522	33,954,653 (87.52%)	44.26	91.76

Note: The percentage in brackets is the percentage of mapped reads in the total reads.

Table S4. Primer list of RT-qPCR

Gene name	Forward primer	Reverse primer
DcActin1	CGGTATTGTGTTGGACTCTGGTGAT	CAGCAAGGTCAAGACGGAGTATGG
DCAR_012429	ACTAATGGTGTCTCGGGCG	CAGAGCAATCTCCATGCCAG
DCAR_016234	AGAGCATGGGTATCGAAGGTG	CCAGGCTCTCTAACACCATTG
DCAR_030523	CGGTGATGAGCTTCTTTCGG	CCTCAGCAGAAGGGTTGGC
DCAR_003708	TCCAATCACACCAAGGCAGG	TTCCCTCTGAGCCCATCAGT
DCAR_026162	GCCTTGACTCCTACGGACAG	CCATTCACCACAGCTTGCC
DCAR_018164	GGTTTCGGGACCTTGTTGGA	CAGACCAGGACGCAAAGTCA
